# Root exudates contribute to belowground ecosystem hotspots: A review

**DOI:** 10.3389/fmicb.2022.937940

**Published:** 2022-10-05

**Authors:** Wenming Ma, Sihong Tang, Zhuoma Dengzeng, Dong Zhang, Ting Zhang, Xiangli Ma

**Affiliations:** Institute of Qinghai-Tibetan Plateau, Southwest Minzu University, Chengdu, China

**Keywords:** plant nutrition, SOC, allelopathy, soil microorganism, aggregates

## Abstract

Root exudates are an essential carrier for material cycling, energy exchange, and information transfer between the belowground parts of plants and the soil. We synthesize current properties and regulators of root exudates and their role in the belowground ecosystem as substances cycle and signal regulation. We discussed the composition and amount of root exudates and their production mechanism, indicating that plant species, growth stage, environmental factors, and microorganisms are primary influence factors. The specific mechanisms by which root secretions mobilize the soil nutrients were summarized. First, plants improve the nutrient status of the soil by releasing organic acids for acidification and chelation. Then, root exudates accelerated the SOC turnover due to their dual impacts, forming and destabilizing aggregates and MASOC. Eventually, root exudates mediate the plant–plant interaction and plant–microbe interaction. Additionally, a summary of the current collection methods of root exudates is presented.

## Introduction

Plants input a range of compounds to soil *via* root (especially fine toot) at their growth stage. Such compounds are known as root exudates. The root exudates entering the soil can change the physicochemical properties of the soil and such part of the soil is known rhizosphere (Gregory, 2008). German microbiologist Lorenz Hiltner first proposed the concept of the rhizosphere in 1904, when he found a higher abundance of microbe in the rhizosphere than in the bulk soils (Yin et al., 2018). Root exudates are normal and positive physiological phenomena of the root system, which is an inherent physiological function of the root system and an adaptive mechanism developed by the plant due to long-term adaptation to the external environment (Longchi et al., 2002). In recent years, researchers have studied the release pathways of root exudates. The study of the membrane protein family that mediates the release of root exudates becomes a hot spot. In recent years, a great deal of research has been carried out on the composition of root exudates and the mechanism by which they are synthesized and released (Marschner, 1995; Jones, 1998; Carvalhais et al., 2011; Wang and Lambers, 2020).

The presence of root exudates significantly affects the physical and chemical properties of rhizosphere soil (Hinsinger et al., 2005; Bais et al., 2006). Plants actively regulate the rhizosphere environment by releasing root exudates into the soil, thus making the rhizosphere one of the most dynamic and vibrant interfaces in terrestrial ecosystems, becoming a hotspot for plant-soil-microbe interactions and material cycling (Hinsinger et al., 2005), which plays a vital role in material cycling in terrestrial ecosystems as well.

Since soil is a mixture of water, air, minerals, organic matter, and living organisms, root exudates present at the interface between root and soil will have an initial effect on the physical and chemical properties of the soil as well as the microorganisms and plants that exist in the soil (Brevik et al., 2015; Giri et al., 2018). This paper focuses on how root exudates being released into the rhizosphere and lay emphasis on their fate in the soil.

## The properties of root exudates

### Composition of root exudates

The composition of root exudates is complex and varied which, include three fractions, namely diffusates, secretions, and excretion. Root diffusates are low molecular weight organic compounds that are passively diffused out of root cells. Root secretions refer specifically to organic compounds of a particular physiological role, they play a crucial role in signaling, nutrient transport and stress resistance. Root excretions include mucilage, colloid, and lysate, derived from root cell bio metabolism (Neumann et al., 2000). At the same time, it has also been argued that root exudates consist only of soluble substances released by plant roots (Yin et al., 2018). Usually, root exudates have been broadly classified into three categories based on their molecular masses for research convenience. They are LMW (low-molecular-weight) compounds, HMW (high-molecular-weight) compounds and ions. The low molecular weight root exudates mainly consist of sugar, amino acids, phenol, etc. The HMW exudates derive mainly from mucilage and extracellular enzymes, where the mucilage consists of polysaccharides and polyuronic acids (Badri and Vivanco, 2009). Plants release these compounds to change the rhizosphere’s physical, chemical or biological properties to improve the nutrient absorption through root systems, which is the key factor regulating the rhizosphere microecological function (Wu et al., 2014).

### Amount of root exudates

An essential component of belowground carbon input to plants is root exudates, accounting for 5–21% of photosynthesis products annually (Marschner, 1995). Nearly 50% of the carbon fixed by soil-grown plants is distributed to the soil, half of this C is retained as root tissue, and the other half is root products (root exudates, border cells, and root debris) (Neumann et al., 2000). Thus, root exudates, although small, are an essential part of the global C-N cycle. And the amount of root exudates varies in different plant species. Many perennial woody and herbaceous plants, with deep and extensive root or rhizome systems belowground, can produce a large amount of root exudates over quite a long time. In addition to the plant species, the cultivar, age of plants, soil properties, and stress factors, they all have varying degression of effects on the amount of root exudates (Uren, 2007).

### Mechanism of root exudates release

The release of root exudates is essentially transmembrane transport. Based on the mode of transportation, the release mechanism of root exudates is divided into two types, passive transport and active transport (Bertin et al., 2003). Passive transport further includes three pathways. The very typical one is to diffuse through the membrane (diffusion), which releases low-molecular-weight (LWM) sugar, amino acids, carboxylic acids, and phenolics. And the whole process is driven by different concentrations between the cytoplasm of root cells and the rhizosphere, along with the membrane permeability and polarity of the compound that was about to exudate (Badri and Vivanco, 2009). However, when faced with specific stress such as nutrition efficiency or warming/Al toxicity, specific carboxylates (e.g., citrate, malate, and oxalate), used to exudate in high concentrations normally, are not able to diffuse through the root membrane. When diffusion is restrained, the ion channels (protein) located in the cell membrane can function (Dreyer et al., 2012). And there are two types of ion channels, including Slow Anion Channels (SLACs) and QUick Anion Channels (QUACs). The former needs several seconds to be activated, while the latter only requires a few milliseconds (Dreyer et al., 2012). The third mode of transport is vesicle transport, where the substances are HMW compounds, such as mucilage, polysaccharides, proteins. In addition to the three types of passive transport (diffusion, ion channels, and vesicle transport), root exudates can also travel in an active transport *via* proteins located in the root plasmatic membrane (Baetz and Martinoia, 2014). The root exudates released *via* proteins are mainly primary and secondary metabolites. Primary metabolites refer to various chemical substances released by the root system to maintain plant growth and development in the face of external stress, while secondary metabolites do not directly contribute to plant growth and development, but rather indirectly improve the plants’ adaption to adversity by altering their surround environment (Neumann et al., 2000; Badri and Vivanco, 2009). Root exudates from the non-metabolic pathway are not regulated by plant metabolism, mainly including intercellular permeates, decomposition products, and inclusions of root cells (Uren, 2007). Transporters are a class of proteins with transmembrane transport functions. These proteins are usually embedded in biofilms in the form of oligomerization to form unique channel structures. Transporters can be divided into two categories according to whether they have an ATP-binding domain. One is the ATP-binding cassette (ABC), which has an ATP-binding domain and obtains energy through hydrolysis of ATP in the transport process (Loyola-Vargas et al., 2007), and the H^+^/Na^+^ gradient-dependent transporter, which consists of Multidrug and toxic compound extrusion family (MATE) (Kang et al., 2011), the major facilitator superfamily (MFS) (Reddy et al., 2012) and the aluminum-activated malate transporter family (Weston et al., 2012).

**FIGURE 1:**
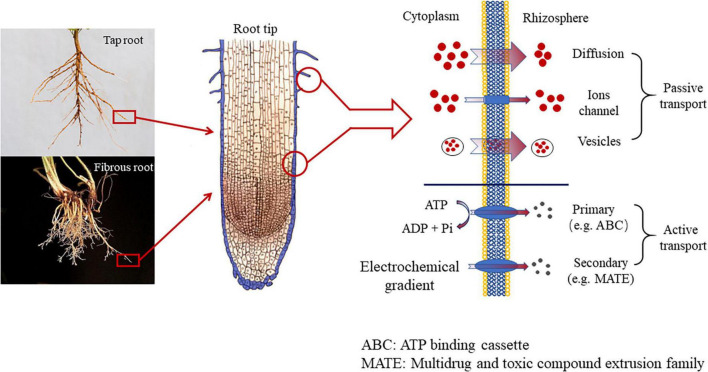
The mechanism of root exudation.

**FIGURE 2:**
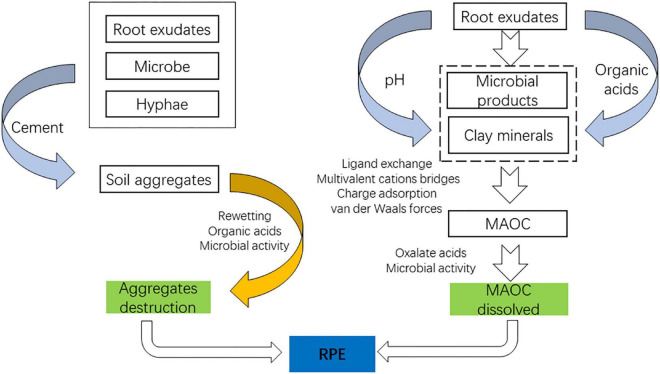
Pathways of root exudates regulating SOC.

**TABLE 1:** The general composition of the root exudates.

Root exudates effects		Example
Interspecific facilitation *via* nutrient mobilization driven by root exudates	Mobilization and absorption of insoluble P in soils	In faba bean/maize intercropping, the roots of faba bean released carboxylates or proton to promote P uptake of faba bean and maize.
		In maize/chickpea or wheat/chickpea intercropping, acid phosphatase released from chickpea roots promotes the P uptake of different crops.
	Improvement of nodulation and nitrogen fixation of legumes	Root exudates from maize include flavonoids improving the nodulation and nitrogen fixation of faba bean in faba bean/maize intercropping.
	Mobilization and absorption of Fe	In peanut/maize intercropping, phytosiderophores secreted by maize roots help to solubilize Fe (III) in the form of Fe (III)-PS complexes, which reduced to Fe (II) and taken up by the peanut.
Root exudates reducing heavy metal toxicity and promoting the neighbor plants	Improvement of tolerance to Al3+ toxicity	Malate, citrate, and oxalate secretion has been demonstrated to chelate free Al3+ and mediate the Al tolerance of cereals including wheat, barley, and rice.
	The heavy metal uptake by crops is decreased in heavy metal hyperaccumulators/crops intercropping system	Intercropping generally enhances low molecular weight organic acid secretion from the roots of nightshade to construct chelation with Cd2+, the heavy metal uptake by hyperaccumulators is increased and the heavy metal uptake by crops is decreased under nightshade/maize intercropping.
		Intercropping of *Sonchus asper*/*Zea mays* changes the types and amount of organic acids in root exudation, which increases Cd uptake and accumulation in *S. asper* but inhibits Cd accumulation in maize.
		Intercropping of *Sonchus asper*/*Zea mays* resulted in the increase of the citric acid and oxalic acid secreted by *S. asper*, which increased the uptake and accumulation of Pb in *S. asper*.
Interspecific facilitation driven by root exudates regulating microbial community	Infection of soil-borne diseases *via* regulating microbial community	Intercropping with aerobic rice suppressed *Fusarium* wilt in watermelon by reducing the density *Fusarium oxysporum.*
		Antimicrobe compounds secreted by maize resulted in the inhibition of *Phytophthora capsici* disease in maize and pepper intercropping systems.
Plant interspecific allelopathy mediated by root exudates	Quinones allelochemicals	The napthoquinone and juglone are produced by the root of black walnut, which can inhibit the growth of neighboring plants.
	Flavonoids allelochemicals	Rice roots secrete diterpenoid lactones, cyclohexanone, and flavonoids to suppress weeds.
	Benzoxazinoids allelochemicals	DIMBOA allelochemicals released by wheat roots inhibit weeds.
	Alleviation of autotoxicity	In continuous Chinese fir monocultures, Chinese fir trees produce and release a novel allelochemical cyclic dipeptide into the soil, resulting in autotoxicity, if Chinese fir is planted with *Michelia figo* the inhibition will be alleviated.

## Factors affecting root exudates

The composition and quality of root exudates are influenced by the plant’s genotype so different species of plants have innate differences in the composition and quality of their root exudates (Hu et al., 2018). However, in addition to innate genotypic influences, the environment in which the plant grows can also affect the composition and quality of root exudates. Species differences (Naveed et al., 2017), phenophase, root morphology (Proctor and He, 2017), soil properties (Vance et al., 2003), and microorganisms (Bais et al., 2006) all affect root exudates.

### Biological factors

The root exudates vary significantly from plant species both in quantity and quality. Naveed et al. (2017) measured the composition of root exudates from barely (*Hordeum vulgare* L. cv. Optic) and maize (*Zea mays* L. cv. Freya). Barley had root exudates of 10.8% amino acids, 47.2% organic acids, 2.8% fatty acids, 15% sugars, sugar acids, and sugar alcohols, and 15% phosphoric acid. In comparison, maize had 5.7% amino acids, 27.8% organic acids, 13% fatty acids, 17.8% sugars, sugar acids, and sugar alcohols, 24% phosphoric acid, and 9.6% urea. In addition, the cultivars also affect the root exudates pattern. Du et al. (2020) quantified low-molecular-weight organic acids released from the roots of two rice cultivars and found significant variation between cultivars, high Phthalic acid esters accumulating rice cultivar exudates twice as possible much oxalic acid, citric acid, and malonic acid as another one. In addition to plants with different species, the quantity and quality of root exudates vary considerably in the same plant at different growth stages. Zhao et al. (2021) found significant differences in Arabidopsis root exudates during slow and fast growth stages. But there was no difference in the composition of substance species in root exudates. This difference was caused by the different absolute amounts and relative abundances of each compound. Fast-growing stage plants released a higher quantity (cumulative abundance) of sugars, sugar alcohols, organic acids, amino acids, amides/amines, and unknown compounds. In contrast, the slow-growing stage plant’s root exudates had a higher relative abundance of sugars, sugar alcohols, and organic acids. Phosphorus deficiency caused white lupine to release more malic acid in the early stages of cluster root formation and less malic acid and more citric acid in the later stages of cluster root formation (Neumann et al., 1999). Root exudates act on the surrounding environment of the root system to produce rhizosphere effects. At the same time, rhizosphere microorganisms tend to colonize the plant root system and play an important role in modifying and limiting root exudates through their metabolic activities by decomposing and transforming root exudates and abscissions (Sasse et al., 2018). The root exudates have increased a lot due to the presence of microorganisms. Prikryl and Vankura (1980) found that wheat root exudates are twice as high under natural conditions as under sterilized conditions, suggesting that rhizosphere microorganisms stimulated the release of root exudates. Graham (1988) indicated that rhizosphere microorganisms could alter plants’ physiological and biochemical processes by changing the rhizosphere nutrient status and plant hormone content, thus affecting the composition and amount of root exudates. Microorganisms are involved in the decomposition and mineralization of soil organic matter, increasing the availability of soil nutrients and promoting plant growth. The plant’s vigorous growth encourages the increase of root exudates (Marschner, 1995; Pinton et al., 2007; Liang et al., 2017). And, rhizosphere microorganisms can selectively utilize specific components of root exudates, thus changing the original composition and ratio of root exudates.

### Chemical factors

Soil nutrients and heavy metal stress are the main chemical factors affecting root exudates. The plant’s growth environment affects the plant’s physiological state all the time, and root exudates act as a competitive functional trait for fine roots to help the plant adapt itself to environmental changes to obtain nutrients and resist stresses (Sun et al., 2021). Soil nutrient deficiencies induce an increase in the release of primary metabolites. For certain plants, P deficiency can significantly alter the composition of root exudates. Increased content of organic acids, protons, acid phosphatase and carboxylates in root exudates improves the soil P bioavailability (Dong et al., 2020). While lower root exudates of carboxylates, sugar, and amino acids have been observed in nitrogen-deficient legumes (Haase et al., 2007); Fe-deficiency and K-deficiency tend to improve the amount of sugar and organic acids in root exudates of maize (Carvalhais et al., 2011). In addition to poor soil nutrient conditions, heavy metal stress can also alter root exudates. The toxic effects of aluminum on plants mainly manifested in affecting the elongation and division of root cells, damaging the structure and function of cell membranes, interfering with the uptake and metabolism to mineral elements, and impairing the activity of enzymes, etc. Under aluminum stress, releasing lots of organic acids is the main mechanism by which some plants resist aluminum toxicity (Pellet et al., 1995; Jones, 1998). Large amounts of organic acids would chelate directly with aluminum in the rhizosphere, reducing the aluminum entering the cells and lowering the toxicity of aluminum (Magalhaes et al., 2007). Citric acid, malic acid, and oxalic acid are the main organic acids associated with aluminum stress (Longchi et al., 2002). For instance, maize would release a large amount of organic acid when faced with aluminum stress, reducing the uptake of aluminum ions by maize root tip cells. Al^3+^ induces increased citric and malic acid exudation in maize root, with acid predominating and exudation being two to four times greater than malic acid (Jorge and Arruda, 1997). Besides the release of organic acids, the mucilage released by the root tips of plants can effectively prevent aluminum ions from entering the cells of the root meristem and enhance the aluminum tolerance of plants (Miyasaka and Hawes, 2001).

### Physical factors

Soil physical properties are also an important factor affecting root exudates. For instance, drought or flood can harm plants and root exudates will be one way of coping with this environmental stress. Drought increases the soil particle’s mechanical pressure on the roots and stimulates the root exudates (Marschner, 1995). The increased release of mucilage by the root system allows soil particles to cover the root surface and form a root sheath, especially for the graminaceous plants, to reduce the water loss (McCully, 1999). Also, the ambient temperature affects the root exudates. Root exudates are affected by temperature both in quality and quantity; any sudden temperature changes would stimulate root exudates (Pinton et al., 2007). Research has shown that the amino acid content of strawberry root exudations is much higher at a lower temperature (5 ∼ 10°C) than at a higher temperature (10 ∼ 15°C). It has been found that warming significantly alters fine root exudates and carbon fluxes (Qiao et al., 2014). Ma (2020) measured the carbon and nitrogen inputs to root exudates of *Sibiraea angustata* (Rehd.) Hand.-Mazz under artificially warmed treatments at the eastern margin of the Tibetan plateau, finding that the elevated temperature significantly increased the rate of carbon and nitrogen input to root exudations by 14.0–69.1% and 15.3–70.2%, respectively, while the flux of carbon and nitrogen of the root exudations increased significantly by 57.2 and 46.9%. This phenomenon may be related to the diffusion-mediated release of root exudates that the temperature variation could give rise to a disturbance in the membrane (Pramanik et al., 2000). Like temperature, CO_2_ and light intensity also affect root exudates since a large proportion of the organic carbon from root exudates is fixed by photosynthesis (Uren, 2007). It’s generally accepted that elevated CO_2_ concentrations can increase plant biomass, along with the amounts of root exudates (Rogers et al., 1994). A meta-analysis conducted by Dong et al. (2020) indicated that elevated CO_2_ concentration increased the efflux rates of soluble sugars, carboxylates, and citrates by 47, 111, and 16%, respectively. And this phenomenon may be attributed to the increased root biomass. According to the experiment conducted by Li et al. (2020), Scots pine (*Pinus sylvestris*) roots exudate more C at low light. Overall, the plant’s physiological state is affected by the environment, which in turn affects the root exudates.

## The function of root exudates

### Improving soil nutrition deficiencies

Plant roots and their exudates improve the soil nutrient status to ensure healthy plant growth. Cluster rooting is a manifestation of plant adaptation to poor soils and is described as a large number of defined branching roots formed along the primary root (Shane and Lambers, 2005). Cluster roots can increase the root surface area by more than 140 times and the volume of soil roots explore by 300 times (Lamont, 2003); thus, it directly expands the absorption area of the plant root system. It also indirectly enhances the plant’s uptake of immobile nutrients by releasing more organic acids (Dinkelaker et al., 1989). The cluster roots’ origin, growth, and release of substances are controlled by many environmental factors (Lamont, 2003). Among these factors, P and Fe deficiencies are the most important. White lupine is one such typical plant that grows in Australia and in soils with low P availability. To deal with P deficiency, lupine developed thick clusters of roots. With a larger surface area, these roots release larger amounts of carboxylates (citric acid), and the number of carboxylates varies with the plant’s age and phosphorus availability (Dinkelaker et al., 1989; Wasaki et al., 2005). In addition to organic acid, the plant roots also release phosphatase and phytase to improve P deficiency (Wang and Lambers, 2020). Low molecular weight organic acids in root exudates, such as citric acid, malic acid, malonic acid, and oxalic acid, are primarily present in the soil in the form of fully dissociated anions because of the soil pH and organic acid dissociation properties (Jones et al., 2003). And the specific mechanism of mobilizing phosphorus is as follows (Tian et al., 2019; Wang and Lambers, 2020):

#### Competition of absorption sites

Organic acids, which are present in the soil in the form of organic anions, can occupy sorption sites for soil minerals. Organic anions can replace phosphorus adsorbed on the surface of Fe/Al oxides or compete with phosphate ions for binding sites of soil particles to reduce the adsorption and fixation of phosphate in the soil. Therefore, the bioavailability of phosphorus in the soil was increased.

#### Change in surface charge

Organic anions change the charge on the surface of adsorbents such as Fe/Al oxides in the soil through complexation reactions, thereby reducing their fixation by phosphate adsorption. Under normal pH conditions, Fe/Al oxides in variable-charge soils are generally positively charged. The complexation with organic anions can change the charge on the surface of these adsorbents. After the adsorption of organic anions, the positive surface charge of rhizosphere soil decreased, thus reducing the adsorption and fixation of phosphate and improving the bioavailability of soil phosphorus.

#### Mineral dissolution

Acid solubilization and complexation together promote the dissolution of minerals. Plant roots releasing protons resulted in a significant decrease in rhizosphere pH, promoting the dissolution of insoluble phosphorous-bearing minerals or compounds. The complexation reaction between organic acids/organic anions and metal ions such as Fe^2+^, Al^3+^, and Ca^2+^ causes phosphorus-bearing compounds or minerals to dissolve and thus mobilize phosphorus in the soil. Second, organic anions accelerate the dissolution of minerals by affecting the saturation state of the solution to the minerals. Third, organic anions affect the dissolution rate of minerals by changing the form of ions in the solution. In addition, organic acids can dissolve Fe/Al oxides, calcium carbonate, and other minerals that can adsorb soil phosphorus, thus significantly increasing soil phosphorus mobility (Wang et al., 2017).

#### Assembly microorganisms

Root exudates provide a carbon source for soil microorganisms, and plants recruit different microorganisms to the root zone under the guidance of root exudates (Sasse et al., 2018). The recruited phosphorus-releasing microorganisms can promote the release of inorganic phosphorus and the decomposition of organic phosphorus in the soil by releasing organic acids and phosphatase, respectively, to improve phosphorus availability in the soil (Avis et al., 2008). For instance, ectomycorrhizal hyphae can release oxalic acid and some enzymes into the soil to mobilize soil inorganic and organic phosphorus (Smith et al., 2018). Besides microorganisms, plant roots can also release acid phosphatase to catalyze organophosphorus’s hydrolysis and release inorganic phosphate ions (Tian et al., 2019).

In addition to improving the P availability in soil, root exudates can also affect two other vital elemental nutrients that are primarily required by plants (i.e., nitrogen and potassium). When the plant is deficient in nitrogen, the overall growth is limited, and the plant becomes short or may even stop growing. It was found that specific root exudates released by certain plants can inhibit nitrification (Coskun et al., 2017). Such root exudates are known as biological nitrification inhibitors (BNIs). They can selectively inhibit the activities of nitrifying bacteria in the soil, thus slowing down the reaction rate of conversion of ammonium to nitrate in the soil. And five BNIs have been identified from plants: brachialactone (from brachyurum), sorghum quinone and MHPP methyl p-hydroxyphenylpropionate (from sorghum), 1,9-anisidine (from rice), and sakuranetin (a hydrophilic flavonoid), of which sorghum quinone is well-studied, it is produced only in root hairs and exuded from the root hair tips. BNIs have also been found in wheat root exudates but not yet in maize. Moreover, the site of BNIs release and the mechanism of its production still need more research.

Potassium is of great benefit to plant metabolism. However, fewer studies have been conducted on the influence of root exudates on the biological activity of potassium. Organic acids have a solubilizing effect on minerals. Thus, root exudates containing organic acids can form a potassium-releasing effect on potassium-bearing minerals. Song and Huang (1988) experimented on the impact of organic acids on the dissolution of potassium-bearing minerals, revealing that the rate of potassium release depends on the nature of organic acids and the formation sequence of potassium-bearing minerals.

Besides the three nutrients mentioned above, plants need specific trace elements for their growth and development. As a biologically essential element, Fe is vital for the growth and development of all organisms. Despite the presence of large amounts of Fe in the earth’s crust, in most cases (especially under aerobic conditions), Fe exists mainly in the trivalent state (Fe^3+^), fixed in insoluble hydroxides and oxides (e.g., ferric hydroxide, ferric oxide, and ferric tetroxide), and is hardly available to live organisms (Monsen, 1988). To make full use of Fe, several species of microorganisms (bacteria and fungi) and plants (gram crops) have gradually acquired the ability to synthesize and release siderophore during a long evolutionary process. Generally, Fe uptake mechanisms in plants are divided into two strategies (Neumann et al., 2000).

##### Strategy I plants

The mechanism of strategy I plants is characterized by the reduction of ferryl and the uptake of divalent iron, which is common to both dicotyledonous and non-gramineous plants. In well-ventilated soil, Fe is mainly present as insoluble trivalent iron (Fe^3+^) oxides or hydroxides, the effectiveness of which increases significantly with decreasing soil solution pH. Therefore, the Fe bioavailability is highly dependent on changes in soil solution pH (Olsen et al., 1981). Strategy I plants improve iron uptake by roots through the change in soil pH.

The first mechanism to mobilize Fe is the rhizosphere acidification caused by protons (H^+^) release. The leading cause of rhizosphere acidification is the unbalanced uptake of anions and cations by the root system. Plants regulate their net charge balance by releasing protons and bicarbonate from the root system, directly controlling the rhizosphere pH (Gregory, 2008). The release of protons is mediated by H^+^-ATPases (AHA) activity located in the plasma membrane. Usually, the strategy I plants can specifically induce H^+^-ATPases (AHA) in roots at the early stages of Fe deficiency and increase Fe mobility by releasing protons to acidify the rhizosphere (Santi and Schmidt, 2009). Also, the organic anions released from roots can have a synergistic effect on rhizosphere acidification. When organic anions are released from the root to the rhizosphere, the net charge balance of plants is disrupted. Therefore, to restore balance, plants take up OH^–^ or release H^+^, along with organic anions (Gregory, 2008), which slightly contributes to rhizosphere acidification.

The second mechanism of improving Fe availability is the chelation of insoluble Fe by released organic acids. In addition to protons, plants also actively release LMW organic compounds at the rhizosphere to enhance rhizosphere Fe availability, mainly including organic acids, phenols, and flavonoids (Marschner, 1995). Organic acids are of particular importance among these root exudates, such as citric acid, malic acid, and oxalic acid, which promote Fe uptake mainly through chelation by mobilizing insoluble Fe or by acidifying rhizosphere (Jones and Darrah, 1994).

In addition to chelation and acidification, a part of low-molecular-weight compounds also can be used to increase the mobility of Fe by reducing trivalent iron in soil to divalent iron with higher solubility (Brumbarova et al., 2015). For example, dicotyledonous plants, such as *Beta vulgaris*, are deficient in Fe, and the flavonoids in the root exudates are highly reductive (Sisó-Terraza et al., 2016).

##### Strategy II plants

Strategy II plants are plants that release phytosiderophore (PS) to enhance rhizosphere Fe’s mobility (Neumann et al., 2000). Takagi et al. (1984) found that the Fe uptake by graminaceous plants is enhanced by the siderophore release, which has a solid complexing capacity for Fe^3+^. These compounds have high specificity and affinity for Fe and can chelate trivalent iron ions, thus enhancing Fe mobility (Ma, 2005). Moreover, the complexation of Fe^3+^ by PS is not exclusive, which can also be complex with other trace metal elements (e.g., Cu, Zn, Mn, Co, and Ni), of which bioavailability are enhanced (Neumann et al., 2000).

The PS is biosynthesized in the root cell and transported to the rhizosphere through a vesicle or anion channel, where it mobilizes the Fe and other metal anions such as Mn^2+^, Zn^2+^, Cu^2+^ (Neumann et al., 2000). And the PS from plants is strongly chelated with Fe^3+^; thus, the chelation efficiency is unaffected by the amounts of Ca^2+^ and Mg^2+^ in the soil and much less affected by pH than that of strategy I plants. In the soil, this chelate moves to the root surface and is absorbed by a specific transfer system on the plasma membrane, and then the PS is released back into the soil, while Fe^3+^ is absorbed by the plant (Ahmed and Holmström, 2014).

Although only graminaceous plants have been found to release PS, non-graminaceous plants can assemble the siderophores-releasing microbes in the rhizosphere. The LMW organic compounds produced under Fe deficiency (e.g., phenolic substances) can indirectly increase the availability of rhizosphere Fe by altering the microbial community structure. Jin et al. (2006) found that phenolic compounds produced by red clover roots under Fe-deficient conditions have dual impacts on the rhizosphere microbe. On the one hand, they inhibited the growth of phenol-sensitive microorganisms through their antimicrobial function. On the other hand, they promoted the development of phenol-tolerant microorganisms as a carbon source. These two effects lead to the assembly of siderophore-releasing microorganisms in the rhizosphere. The microbial siderophores can dissolve insoluble Fe oxides in the rhizosphere soil by chelation, and then plants readily absorb the Fe (III)-siderophore complex (Ahmed and Holmström, 2014).

### Affect SOC turnover

Recent studies have shown that living roots and microbes are important drivers of increased organic carbon stocks in tops soil and subsoil. Firstly, the binding of microbial residues to the mineral surface contributes to SOC accumulation, and microbial residues are derived from a relatively small amount of microbial biomass, accounting for approximately 50% or more of the stable SOC. Secondly, in addition to the biological residues mentioned above, charged unstable organic carbon compounds/molecules may also enter the direct sorption pathway on mineral surfaces or partially contribute to the desorption of natural SOM. The main source of these charged organic compounds is organic acids in compounds. Another stabilization mechanism for SOC is then physical confinement, i.e., part of the degraded plant residue, originating from the root system, is stabilized in the soil aggregates. A significant amount of soil carbon input comes from the belowground plant process (Sokol et al., 2019). Photosynthetically fixed carbon is deposited within the rhizosphere primarily as root biomass, exudates, and microbial biomass, as soil SOM. And it has been recently suggested that there is a paradox between the stability and instability of SOC due to plant roots interfering, including exudation. Multiple factors influence the effect of root exudates on SOC stabilization or SOC replenishment. Root exudates contain the majority of non-volatile rhizodeposits and an abundance of soluble organic compounds, such as sugar, amino acids, and organic acids. Both low-molecular weight root exudates and mucilages can be used as a carbon source by the microbial community. Various studies have examined the role of important root exudate compounds in SOC stabilization. For example Keiluweit et al. (2015) used exogenous applications of glucose and oxalic acid (compounds frequently present in root secretions) to study CO_2_ emissions induced by forest soil microbial communities. Their analysis showed that the addition of oxalic acid induced a more pronounced positive initiation effect compared to glucose. Similarly, Luo et al. (2014) tested the respiration rates of soil samples from different habitats after treatment with glucose, citric ad oxalic acids, but with conflicting results. The glucose amendments have the highest respiration rate, while the oxalate amendments do not produce a positive priming effect in the different biological environments. Some researchers have argued that the stability of organic carbon added to the soils is largely influenced by the nature and properties of the soils and ecosystem, and is less dependent upon the chemistry of the added compounds (Schmidt et al., 2011), which might account for the phenomenon that the same components showed contrasting results in term of SOC stabilization. For instance, organic acids, such as oxalic acid can form stable SOC components by binding to Al/Fe oxides (Yuan et al., 2020), what’s more, they can also demineralize existing SOC pools (Keiluweit et al., 2015).

#### Forest

Forest can be divided into five main biomes: boreal, polar, temperate, subtropical, and tropical. Of these five biomes, tropical forests account for 45% of the total forest land (Nesha et al., 2021). Quantitative data on soil organic carbon content in the top 100 cm of tropical, temperate, and boreal coniferous forest soils show that the soil organic carbon content of tropical coniferous forests is 214,435 Gt, while that of temperate and boreal coniferous forests is 153, 195, and 338 Gt, respectively (Lorenz and Lal, 2009).

#### Grasslands

Compare to forests, grassland is a nature reserve for organic carbon, with an organic carbon stock of about 439 G (Montanarella et al., 2015). Grass secretes excessive amounts of organic compounds, organic acids, and amino acids that are relatively abundant in the form. High species richness showed a high organic carbon accumulation of grassland in an experiment on grassland biodiversity (Lange et al., 2015). In addition, grassland vegetation densities with higher species richness also resulted in lower evaporation rates, which mitigated the effects of climate on SOC decomposition (Poirier et al., 2018).

#### Shrublands

Previous studies have shown that aboveground litter is an important source of SOC in the surface soil, while root turnover and rhizosphere deposition are the main sources of SOC in the deep soil (Tamura and Tharayil, 2014). Many studies have confirmed that shrub roots are more developed than herbs, and their carbon sequestration ability will significantly affect the carbon storage of the whole alpine grassland ecosystem (Zhou et al., 2017). Due to the incorporation of SOC-rich material, dead tissues, or exudates into deep soils, causes high changes in deep SOC stock in shrubland (Li et al., 2021). The fine roots of herbaceous plants can encapsulate soil organic matter, increase soil organic carbon content, and increase litter input biomass and root exudates (Zhang et al., 2022). A previous study found for the 0- to 200-cm soil layer, SOC stocks were significantly lower in shrubland than in either cropland or grassland (Wang et al., 2016).

The decomposition process of organic matter entering the soil can be divided into two stages depending on the rate of decomposition: the first stage is the rapid decomposition of soluble and easily decomposable matter entering the soil, which takes between 3 and 6 months depending on the soil conditions; the second stage is the slow decomposition of substances that are difficult to decompose (Zhang et al., 2016). The quality and quantity of root exudates are gene-based, modified by various plant species, but the further transformation of root exudates into the soil is independent of the plant species. Liang et al. (2017) introduce the concept of microbial carbon pump, which refers to the continuous transformation of organic carbon entering the soil from unstable forms to physicochemically protected forms by microorganisms through anabolic metabolism. Since SOC is stabilized by two main mechanisms, the formation of soil aggregates and the formation of mineral-associated soil organic carbon (MASOC). Root exudates possess a great ability to regulate the terrestrial SOC stock. And due to the complex composition of root exudates and the environment surrounding them, root exudates play a dual role in stabilizing SOC.

#### Aggregates

Root exudates accelerate the turnover of aggregates, which on the one hand, promote the formation and stabilization of new aggregates, but on the other hand, can also cause the destruction of old aggregates. Mucilage and polysaccharides usually account for almost 50–70% of root exudates (de Graaff et al., 2010) as a crucial cementing agent, which directly contributes to the formation of aggregates. Traoré et al. (2000) found that mucilage released by maize roots increased the proportion of stable soil agglomerates by 3.8 times after 30 days of laboratory incubation. Moreover, the freshly released mucilage may protect the newly formed aggregates from hydrolysis by sticking to the soil particles. Morel et al. (1991) found that the addition of maize root mucilage delayed the release of soil CO_2_ compared to glucose and rapidly increased the stability of soil aggregates in drying-rewetting soil. Also, root exudates provide microorganisms with a C source, while microorganisms are the most active biological factor affecting the formation and stability of aggregates. Fungi and actinomycetes can form aggregates by mechanically entangling soil particles with each other. In contrast, other microorganisms can form stable aggregates by the cementation of soil particles with their living metabolites (polysaccharides and other organic matter) (Baumert et al., 2018). Soil microorganisms, especially fungi, play an essential role in the formation and stability of macroaggregates (Guggenberger et al., 1999). Bearden and Petersen (2000) found that fungal mycorrhizae effectively improve soil structure and the stability of soil aggregates. Interaction of root border cells with non-pathogenic microorganisms releases unknown compounds that induce mycorrhizal hyphae branching (Sasse et al., 2018). During the formation of soil aggregates, hyphae can play a physical role in the construction of large aggregates. Hyphae can bind microaggregates together by releasing glue-like substances, such as polysaccharides. Afterward, those microaggregates can be entangled by mycelium to form stable large aggregates. Moreover, citric acids, commonly found in root exudates, can promote the formation of Fe/Al (hydro)oxides, while the Fe/Al (hydro)oxides can contribute to the formation and stabilization of soil aggregates as inorganic cement (Yu et al., 2017).

Root exudates can, directly and indirectly, contribute to aggregate formation and stabilization, yet they can somehow lead to soil aggregate destruction. The presence of root exudates and soil water uptake by roots leads to a vigorous drying-rewetting cycle in soils. When dry soil is rapidly wetted, the air within the soil pores is compressed, causing the cracking of aggregates, which releases physically protected organic carbon, being consumed by microorganisms (Denef et al., 2001). In addition to the degradation of soil aggregates, dryness limits the diffusion of soluble organic carbon in the soil, reducing the carbon source for microorganisms. At the same time, some microorganisms with weak drought tolerance were killed by water stress. Still, when the soil was rewetted, microbial activity was rapidly stimulated, and organic carbon was consumed in large quantities (Fierer and Schimel, 2003). Moreover, Non-crystalline Fe/Al oxides, as inorganic cementing substances, are directly involved in the formation and stabilization of aggregates, but oxalic acid in root exudates can dissolve these Fe-Al oxides, leading to the aggregate’s destruction (Li et al., 2018).

#### Mineral-associated organic carbon

The combination of organic carbon and soil mineral composition is considered the most critical stabilization mechanism for soil organic carbon. Root exudates promote MASOC formation by increasing organic matter content and soil mineral availability.

Rhizosphere microorganisms utilize the root exudates for reproduction, and their products (microbial-derived carbon) become the vital source for MASOC. Root exudates input organic carbon into the soil by roots, accounting for up to 40% of the carbon fixed by photosynthesis (Bais et al., 2006). Compared to low-quality litter (high *C*/*N* ratio and phenol/lignin ratio), the root exudates are much closer to the high-quality litter (low *C*/*N* ratio and phenol/lignin ratio) and are readily available to microorganisms. In addition, soil microbial communities are more metabolically efficient in converting living root inputs (*in vivo* microbial conversion pathway) (Sokol et al., 2019). The research shows that the simple and labile plant carbon input (root exudates) will be converted to microbial products more efficiently than complicated and stable plant carbon input (shoot litter and root litter), allowing more simple plant carbon input to remain in the soil and eventually be stabilized by adsorption to soil minerals, which was also known as “entombing effect” (Liang et al., 2017). Thus, root exudates with low molecular weight are more likely to form stable soil organic carbon than plant debris with high molecular weight input. Sokol et al. (2019) found that living root inputs are 2–13 times more efficient than shoot litter input in the formation and stabilization of mineral-associated soil organic carbon (MASOC) and particle organic carbon (POC) pools.

Root exudates also increase the availability of soil minerals, of which the mineral adsorption and saturation are improved. The main clay minerals that contribute to organic carbon stabilization are phyllosilicate minerals (e.g., smectite, kalinite, and illite) and Fe/Al oxide-hydroxides (Pinheiro et al., 2015), whose content in the soil determines the soil carbon sequestration potential. The interaction mechanisms between soil organic carbon and mineral surfaces include ligand exchange, multivalent cation bonding bridges, complexation, relatively weak van der Waals forces, etc. (Wang et al., 2017). Phyllosilicate minerals and Fe/Al oxides have different surface and active absorbing sites, and therefore, their ability to absorb SOC differs.

The adsorption of organic matter by Phyllosilicate minerals is weaker than that of Fe/Al oxides. The adsorption of organic matter by Phyllosilicate minerals is mainly related to their specific surface area. In general, the larger the specific surface area of Phyllosilicate minerals, the stronger their adsorption of organic matter. Wang and Xing (2005) found that the saturation adsorption of humic acid per unit mass of montmorillonite (2: 1) was about twice as much as that of kaolinite (1: 1) under the same conditions. Although the interaction between organic matter and clay mineral surfaces is mainly due to van der Waals forces between the hydrophobic components of organic matter and clay mineral surfaces, the presence of multivalent cations, such as Ca^2+^ and Mg^2+^, on the surface of Phyllosilicate minerals usually acts as an ionic bridge connecting the negatively charged groups in Phyllosilicate minerals and organic matter, facilitating the adsorption of Phyllosilicate minerals onto organic matter (Feng et al., 2005).

Fe/Al oxides are relatively simple soil minerals with dense hydroxyl sites on their surface. Strong bonding occurs between organic molecules and the inorganic hydroxyl groups on the mineral’s surface, known as ligand exchange (Lalonde et al., 2012). And the organic acids in the root exudates enhance the soil minerals availability and promote the formation of SRO minerals, which increase the ability of the carbon sequestration. Yu et al. (2017) find that the presence of living roots allowed the soil to release more than twice the amount of Fe/Al with or without fertilization. Living roots also significantly increased the concentration of SRO minerals. Yu et al. (2017) also showed by adding root exudates (citric acid) to the soil colloid that root exudates drive the transformation of Fe in the soil colloid from goethite to ferrihydrite, while SRO minerals have a larger specific surface area and variable charge, allowing it to stabilize organic carbon. Similarly, oxalic acid, another common organic acid found in root exudates, has a similar effect. Cheah et al. (2003) found that the synergistic action of oxalic acid and siderophore in a two-ligand system accelerates the dissolution of the goethite. Li et al. (2006) found that organic acids promote aluminum release from variable charged soil in terms of rate and quantity, while oxalic acid has a more vital ability to release aluminum than other carboxylic acids (i.e., citric acid, malic acid, and lactic acid). To verify the strong retention of C by both Al and Fe minerals, Yu et al. (2017) designed isotopically labeled experiments in combination with NanoSIMS observations. After labeled ^13^C was added to the soil for 24 h incubation, NanoSIMS images showed significant enrichment of newly added ^13^C with ^27^Al^16^O^–^ and ^56^Fe^16^O^–^. And new SRO minerals formed under the promotion of root exudates can adsorb on the surface of sub aggregates to promote the formation of larger aggregates (Zhou et al., 2013). Also, the interaction of Phyllosilicate minerals with hydrated Fe/Al oxides also affects the mutual sorption of organic matter. Hydrated metal oxides can combine with Phyllosilicate minerals and organic compounds to form a “clay-metal oxide-organic matter” complex. The permanent negative charge on the surface of Phyllosilicate minerals can develop a classical adsorption interaction with positively charged hydrate metal compounds. It was found that the presence of hydrated Fe/Al oxides (ferrihydrite) in the system promoted the adsorption of organic matter on kaolinite (Saidy et al., 2013). It’s suggested that the positive charge of hydrous iron oxides can neutralize the negative charge on the surface of illite and still have a specific concentration of positive charge, and therefore still have good sorption to organic matter.

The release of root exudates and root uptake can lead to acidification of the rhizosphere, while pH is an essential factor influencing the adsorption of organic matter by soil minerals. Phyllosilicate minerals generally have a net negative charge on their surface, and organic matter molecules are also negatively charged, which should repel each other. Still, soil pH can make a shift in this phenomenon. Changes in system pH can cause the surface charge of Phyllosilicate minerals to become less negative or even turn positive (Sarkar et al., 2018). Soils with a lower pH generally have a higher positive charge, facilitating the sorption of negatively charged organic matter. Soils with higher pH have fewer mineral surface sites for organic matter adsorption and may be selective in their organic matter adsorption. Thus, organic carbon adsorption due to electrostatic attraction may also occur at lower pH and the adsorption mechanism of ligand exchange. Mayes et al. (2012) compared the sorption of soluble organic matter in 213 deep soils from the central and Eastern United States. They found that the lower the soil pH, the greater the sorption of soluble organic carbon in all soils studied. In addition, the maximum sorption of organic matter decreased with increasing soil pH.

However, root exudates also act as a double-edged sword for the formation of MAOC, similar to aggregates, which can threaten the formation and stability of MAOC. Root exudates (oxalic acids) can dissolve MAOC already formed, leaving organic carbon exposed to microorganisms (Li et al., 2018). Furthermore, under the culture of root exudates, rhizosphere microorganisms proliferate, and some of them can release microbial siderophores, which can weather Fe minerals. Especially together with the LMW organic acids, a synergistic effect goes on among them, leading to a higher dissolution rate for Fe minerals, which disrupted the protection provided by Fe/Al oxides (Ahmed and Holmström, 2014).

#### Rhizosphere priming effect

Positive RPE driven by root exudates leads to the decomposition of organic matter. Compared to bulk soil, the microbial population in the rhizosphere is 19–32 times larger than in the bulk soil due to the existence of root exudates (Bodelier et al., 1997). The decomposition of SOM may be altered by the rapid turnover of C and microorganisms in the rhizosphere, referred to as the rhizosphere priming effect (RPE). The fundamental mechanism of RPE is the rhizosphere microorganisms demanding nutrients. First, root exudates can stimulate microbial growth and activity using carbon sources, causing synergistic metabolism of SOM (Microbial activation). Second, the uptake of effective soil N by roots promotes the excavation of N from SOM by microorganisms, which results in the decomposition of SOM (Kuzyakov, 2002). The RPE caused by root exudates could accelerate the decomposition of SOM by up to 380% (Cheng et al., 2014). In short, root exudates promoted RPE directly by providing a C source for microorganisms and indirectly by destabilizing organic carbon.

### Mediating plant–microbe and plant–plant interaction

In addition to the direct participation of root exudates in the material cycle of the belowground ecosystem, their exudates of signal substances such as phenols, flavins, and other secondary metabolites can play a role as operators, which makes it a signal bridge between plants and plants and between plants and microorganisms.

#### Root exudates mediate the plant–microbe interaction

##### Root exudates regular the assembly of rhizosphere microbe

Each plant species has a specific rhizosphere microbiota (Deveau et al., 2018). Even the rhizosphere microbial composition varies between genotypes of the same species (Pérez-Jaramillo et al., 2016). The root exudates are the leading cause of this phenomenon. Root exudates contain carbohydrates, amino acids, flavonoids, and phenolic acids, all of which have different effects on inter-rooted microorganisms. Among them, amino acids and carbohydrates provide an effective carbon and nitrogen source for the microorganisms and influence the population distribution of soil microorganisms in terms of quantity and variety. Root exudates regulate the rhizosphere microbe in two main ways: (1) attracting certain species of the microbe and acting as their nutrients, (2) repelling or inhabiting certain species of microbe. For example, Arabidopsis will release malic acid in the root elongation zone to attract *Bacillus subtilis* (Rudrappa et al., 2008). Also, plants produce different types of root exudates in various stages of growth and development, and the specific roles they play are also different. A study by Fangdong et al. (2005) on the rhizosphere microorganisms of roasted tobacco showed that the number of roasted tobacco rhizosphere microorganisms at different growth stages was significantly different. Moreover, root exudates are specific to the action of microorganisms in the rhizosphere. Legumes release flavonoids to attract rhizobia, and the bacteria enter the roots through the gaps between root hairs or epidermal cells (Madsen et al., 2010) and activate the expression of rhizobial nodulation genes, ultimately leading to the rhizobacteria infesting the plant root system to form a symbiotic relationship (Peters et al., 1986). In addition to attracting specific rhizosphere microbe, root exudates inhabit pathogenic bacteria. Such as the phenolic acid in wheat root exudates has antibacterial activity (Wu et al., 2001). The roots of *Ocimum basilicum* L. exude an antimicrobial agent (rosmarinic acid) that may enable itself to resist various microbial infections in the soil (Bais et al., 2002). Also, root exudates can gradually change the plant rhizosphere microbiome by altering soil physicochemical properties. Cluster roots can release large amounts of organic acids that lower soil pH, thereby affecting the growth and colonization of microorganisms. Root exudates not only solubilize phosphate but also modulate rhizosphere microbial regulation of phosphorus uptake (Sasse et al., 2018).

##### Rhizosphere microbe affect the status of plants growth

Certain microorganisms recruited by root exudates can promote plant growth. And the two types of microorganisms involved are called PGPM (plant growth-promoting microorganisms) and BCA (biological control agents); they either directly or indirectly play an important role in promoting plant growth (Avis et al., 2008). PGPM, especially arbuscular mycorrhizal fungi (AMF), can stimulate changes in plant metabolites and produce higher root exudates, which effecting the turnover of the carbon and other nutrients (Ma et al., 2016).

Plant growth-promoting microorganisms are more commonly referred to as plant growth-promoting rhizobacteria, which have absolute numerical superiority among various soil microbiota. Many bacteria species have promoting effects on plant growth. According to the colonization position, PGPR can be divided into iPGPR and ePGPR (Verma et al., 2010), where iPGPR colonizes the tissues or cells of plants to form unique structures (e.g., root noddle) while ePGPR is present around the rhizosphere, promoting plant growth through secondary metabolism and other means. First, PGPR directly enhances the uptake of inorganic nutrients by plants through the mobilization of insoluble nutrients. For instance, many PGPR can release nitrogenase to fix nitrogen in the air for plants and their use (Malik et al., 1997), while some can release extracellular phosphatase to help dissolve organic phosphorus (Wasaki et al., 2005). Also, some PGPR can produce microbial siderophores to chelate Fe^3+^ for their use and that of plants. The microbial siderophores can also compete with rhizosphere pathogens for Fe, inhibiting their growth (Ahmed and Holmström, 2014). Besides the direct chelation with Fe^3+^, PGPR strain GB03 can upregulate the transcription level of FIT1 (Fe-deficiency-induced transcription factor 1) to induce the production of reductase (Fe^3+^) (Zhang et al., 2009). PGPR can also directly promote plant growth by generating certain growth-promoting compounds (e.g., phytohormones, vitamins, amino acids, etc.). Ashrafuzzaman et al. (2009) found that the PGPR strain isolated from rice rhizosphere released IAA (indoleacetic acid), which directs plant stems and roots to respond to light and gravity, respectively, to promote plant growth. The same goes for ethylene, which PGPR promotes plant growth by regulating their concentration at the rhizosphere (Ji et al., 2020). Apart from the direct effects above on plant growth, PGPR can indirectly promote plant growth by releasing metabolites that increase the plant resistance to disease or adversity. Although plants have their defense system for disease or stress, they can play a more significant role with the inspiration of PGPR. Ji et al. (2020) isolated a novel halotolerant strain identified as *Glutamicibacter* sp. (Ydo1). Compared with un-inoculated rice, the inoculated ones have a higher enzyme activity of ACC (1-aminocyclopropane-1-carboxylate) deaminase and a more significant amount of IAA. And the *Glutamicibacter* sp. YD01 inoculation also increases the synthesis of antioxidant enzymes of rice under salt stress, which proves that this PGPR induces the plant defense system against salt stress by the mediation of ethylene and neutralization of ROS.

Analogously, BCA promotes plant growth by fighting off harmful pathogens in various ways. BCA promotes plant resistance to disease in four main ways: antibiosis, parasitism, competition, and induced systemic resistance (Pinton et al., 2007). Antibiosis is the production of specific or broad-spectrum antibacterial compounds by BCA that antagonize pathogenic bacteria to promote disease resistance (Doornbos et al., 2012). For example, the antimicrobial metabolite produced by *Pseudomonas fluorescens*, 2, 4-diacetylmethanetriol, inhibited the pathogen *Sclerotium rolfsii* up to 70% (Asadhi et al., 2013). Parasitism generally occurs between antagonistic microorganisms and pathogenic bacteria, where the antagonistic microorganisms are parasitic on the surface or inside the body of the pathogenic bacteria and affect their normal growth by feeding on the pathogenic bacteria, etc. Trichoderma is prone to parasitic interaction with some pathogens. Moreover, certain actinomycetes can release hydrolytic enzymes to destroy the mycelium or cell wall of pathogens, therefore inhibiting the growth of pathogens (Asadhi et al., 2013). Competition is a strategy whereby beneficial microorganisms compete with pathogenic bacteria for nutritional resources and spatial microsites, thereby and inhibiting the growth of pathogens to reduce disease incidence. For instance, actinomycetes can restrain the growth of pathogens by competing for ecological sites at the rhizosphere, thus reducing the infestation of pathogens, or the same can be achieved by releasing microbial siderophores to compete with pathogens for Fe (Ahmed and Holmström, 2014). Induced systemic resistance (ISR), similar to PGP, is that the MAC inhibits the growth of pathogens directly and induces plants’ resistance mechanism, which enables them to respond to disease and adversity (Doornbos et al., 2012). For example, mycorrhizal fungi can form a symbiosis with the host plant before the pathogens, thus inducing the activation plant defense system and significantly reducing the attack of the pathogens on plants (Schouteden et al., 2015).

#### Mediating the interaction between plants

Root exudates act as signaling agents, which mediate plant–plant interaction. Root exudates can act as direct facilitators or inhibitors for different plants or the same species, depending on their chemical composition and plants’ signaling receptors.

##### Allelopathy

Plant roots release a class of phytotoxic secondary metabolites to inhabit or promote other plants in the vicinity, called allelochemicals, and such a phenomenon is referred to as allelopathy (Perry et al., 2007). Allelochemicals can affect the physiological state of plants and seed germination in many ways. The most common way for allelochemicals to enter the soil is the root exudate from the root hair. It inhibits the growth of the recipient plant by exposing it to cell damage and oxidative damage. Firstly, allelochemicals in the root exudates can lead to changes in the structure of cells. The release of hordenine and gramine from the barley (*Hordeum vulgare*) roots can cause damage to the cells wall, and increase the size and number of vacuoles, disorganization of organelles, and cellular autophagy in the root tip of white mustard (*Sinapis alba* L.) (Liu and Lovett, 1993). Similarly, mustard (*Brassica juncea* L.) roots treated with benzoic acid showed an irregular arrangement of cells and destruction of organelles (Kaur and Kaushik, 2005). Moreover, by accumulating intracellular ROS levels, allelochemicals disrupt membrane integrity and cause structural disintegration of membranous organelles such as chloroplasts and mitochondria within the cell. Afterward, allelochemicals can alter the levels of plant growth regulators or induce imbalances in various plant hormones, thereby inhibiting the growth and development of the plant. And this is related to the majority of phenolic allelochemicals in root exudates, which can stimulate indoleacetic acid (IAA) oxidase activity and inhibit the reaction of peroxidase (POD) with IAA, bound gibberellic acid (GA), or IAA to influence endogenous hormone levels (Yang et al., 2005). Salicylic acid, a common component of root exudates, inhibited ethylene synthesis in pear (*Pyrus communis*) cell suspension cultures (Leslie and Romani, 1988). High concentrations of ferulic acid (2.50 mM) can limit the growth of wheat seedlings by the accumulation of IAA, GA_3_, and cytokinins (CTK), with a parallel increase in abscisic acid (ABA) (Liu and Hu, 2001).

However, low concentrations of allelochemicals are likely to promote plant growth, mainly possible due to the hormesis caused by low concentrations or because the allelochemicals contain nutrients that promote certain plants’ growth (Perveen et al., 2021).

##### Autotoxicity

Autotoxicity is a unique form of allelopathy, an inhibition that occurs between plants within a species. It is a phenomenon of inhibition that occurs when the recipient and donor belong to plants of the same species. Root exudates are how allelochemicals enter the soil, and those allelochemicals can accumulate in the soil. Allelochemicals accumulated in the soil can have harmful effects on the next season’s crop. And plants that undergo autotoxicity are more susceptible to soil-borne diseases (Huang et al., 2013). Cinnamic acid, myristic acid, and fumaric acid in tobacco root exudates accelerate the development of tobacco wilt (Yang et al., 2014). The mechanisms of allelopathy and autotoxicity are the same, but the targets are different. Autotoxic chemicals can promote soil-borne diseases by attracting pathogens. The autotoxic chemicals in tobacco root exudates can act as chemical pheromones to attract pathogens, induce biofilm formation of pathogens, increase the ability of pathogens to colonize the roots, and accelerate the development of tobacco wilt (Li et al., 2017).

## Collection of root exudates

We are much grateful for the kind suggestion. In recent decades, many researchers are constantly improving root exudate collection methods according to actual needs. Smith (1970) developed A technique for field collection of root exudates from mature trees based on a modified air-layering procedure developed by Lyford and Wilson (1996) and Smith (1970). Phillips et al. (2008) proposed a novel *in situ* method for acquiring soluble root exudates in forest soils by describing the advantages and limitations of several commonly employed methods, to measure exudation to their potential adaptability for field use in forest ecosystems (Phillips et al., 2008). The advantage of the method is inexpensive, relatively simple to set up, can be employed throughout the growing season, and requires only a minimal amount of soil disturbance (Phillips et al., 2008). Oburger et al. (2013) compared common sampling techniques with a novel tool (rhizoboxes fitted with a novel *in situ* root exudate collecting tool) for root exudate collection that allows non-destructive and repetitive sampling from soil-grown roots (Oburger et al., 2013), which is a major advance in root exudate sampling. At the same time, Oburger and Jones (2018) addressed critical methodological aspects that need to be considered in the choice of experimental approach, like growth and sampling medium (soil and hydroponic), sterility, sampling location (whole root system, individual root segments) as well as plant age, daytime, re-uptake of metabolites affecting duration and timing of the sampling event and data presentation. And then, they summarized the main analytical approaches on root exudates, including liquid sample analysis, isotope tracking and imaging techniques (Oburger and Jones, 2018). What’s more, Williams et al. (2021) developed root-washing method, which is suitable for ecologically sound root exudate collection and allows accurate measures of exudate quantity and composition (Williams et al., 2021).

### Lab collection

Researchers have invented a range of methods and devices for collecting root exudates based on the ease of operation and practical needs. The collection methods can be divided into two categories depending on whether they are indoors or not. Indoor collection methods (i.e., lab collection) are generally applicable to short-growth plants such as herbs, crops, and other plants. Depending on the environment in which the plants are cultivated, the lab collection methods can be divided into solution culture and substrate culture methods.

#### Solution culture method

The method is to culture plants in solution to obtain a mixture of root exudates solution. The traditional method of solution culture generally refers to hydroponics. In this method, plant roots are rinsed with sterile water and then cultured in ultrapure water with microbial inhibitors for a while; then, the plants are moved, the culture fluid is collected, filtered, and the nutrient ions are removed through a resin to obtain root exudates (Jensen, 1997). Indeed, in addition to using ultrapure water as a culture solution, it is also possible to use other specific nutrient solutions as exogenous stress to obtain root exudates mixtures. For example, Liu et al. (2011) cultivated rice with a nutrient solution containing potassium and calcium. They found that K and Ca nutrients have a regulatory effect on root exudates, affecting rice quality. Hydroponics is a widely used method because it is easy to operate, and the collection process causes minor damage to the root system. Not being carried out in a soil environment allows better control of sterile conditions and avoids the decomposition and use of root exudates by microorganisms. Nevertheless, the solution environment leads to a lack of oxygen in the root system, lack of rhizosphere microorganisms feedback on the root, and absence of mechanical compression of soil particles, factors that directly affect root morphology and the number of root exudates prevent it from reflecting the exudates of the root system under natural conditions (Pétriacq et al., 2017). Thus, hydroponics is used chiefly to collect root exudates in lab cultivation experiments and requires minimal collection time avoiding damage to the plants caused by lack of oxygen. Additionally, some specific nutrient solutions contain high salt content and need to be desalted, quickly affecting the experimental results. With microbial inhibitors added to the solution, the root exudates are heavily diluted, which results in a large volume and low concentration of collected liquid.

#### Soil culture method

The soil culture method can reflect the natural growth of plants and the actual condition of root exudates. The traditional soil culture method involves removing the roots of plants that have been planted in the soil for some time, washing down rhizosphere soil with distilled water, and subsequently obtaining root exudates by centrifugal or filtering (Bhattacharyya et al., 2013). Due to the presence of mechanical compression caused by soil particles, the root system of the plants is highly secretory. Therefore, the amount of root exudates produced per unit of plant dry weight under soil culture conditions is thus likely to be higher than the number of root exudates collected in solution culture conditions (Shi et al., 2011). However, this method requires the extraction of the rhizosphere soil solution, which is difficult to remove soil and microbial influences due to the complex soil composition (Brevik et al., 2015). Besides, it tends to cause damage to the root system during collection, resulting in collected root exudates containing the inclusions and bleeding sap of the root system.

#### Substrate culture method

The traditional substrate collection method uses substrates such as glass beads, quartz sand, agar, vermiculite, or artificial nutrient soil as a substitute for soil to exclude the influence of soil components (Neumann et al., 2009). After a period of incubation in the substrate, the plants are removed and soaked in distilled water or organic solvent to extract the organic matter adhering to the substrate, and then the collected saturated solution is concentrated and filtered to obtain root exudates (Sandnes et al., 2005; Mikutta et al., 2006). Because quartz sand, which is essentially the same size as soil, does not contain the adequate nutrients required for plant growth and has the advantages of being more inert, less likely to react chemically with the root exudates components, better aeration, and some mechanical resistance, it is often used as an alternative to the soil in the collection of root exudations as a substrate for plant growth (Phillips et al., 2011). However, substrates such as glass and quartz sand have poor water retention capacity and lack the mineral elements required for plant growth. This method involves adding a nutrient solution to the substrate to maintain the mineral elements and moisture needed by the plant roots.

To provide an accessible, continuous, dynamical collection of root exudates, some researchers have designed a new series of root exudations collection devices for ongoing root exudates collection based on substrate culture. The continuous root exudate collection system involves transplanting plants into a culture substrate with a root exudation collector at the bottom and injecting the appropriate amount of culture fluid into the container at regular intervals. The organic matter in the root exudation is continuously enriched in the collector, while the culture fluid containing inorganic ions is driven by air bubbles into the culture container of an air-filled pump, thus circulating the liquid in the container and forming an undisturbed-continuous-cycle root exudation device (Tang and Young, 1982). A column of selective adsorption resin is usually connected to the culture fluid drainage port to adsorb organic matter from the aqueous solution. When the root exudates flow through the resin, they are adsorbed on the surface, thus enabling the enrichment and separation of organic matter. After a certain period, the resin is collected, and the root exudates can be eluted from the resin with a suitable eluent.

### Field collection methods

The lab culture allows better control of the sterile conditions and, to a certain extent, avoids the degradation of root exudates by microorganisms, which can be better applied to accurate quantitative analysis (Kuijken et al., 2015). However, this method lacks the influence of rhizosphere microorganisms and the surrounding environment possessed in the field. Therefore, based on the need to study root exudates in the field environment, a modified method for the *in situ* static culture collection of root exudates was devised by Phillips et al. (2008). This method involves rinsing the entire root system of field plants, placing them in a prepared culture device, and burying the device back into the soil. After two equilibration periods of 2–3 days, the device is filled with an appropriate amount of nutrient solution, and after 24 h, the solution is collected. This method effectively mitigates changes in the rhizosphere environment on root exudation and enables the *in situ* collection of root exudates from the plant in the field.

In addition to these methods, some scholars have designed their systems or devices to collect root exudates, depending on the purpose of the study. To collect root exudates from different root segments, the filter paper method was invented, and the details are as follows, prepare chromatographic filter paper washed with methanol and distilled water and dried under aseptic conditions; plant roots were first washed several times in deionized water to remove nutrients attached to the root surface, then the roots were laid flat in a porcelain tray lined with wet filter paper; one pre-treated chromatographic filter paper sheet was placed under each of the roots for collecting exudates, and the rest of the root tissue was covered with wet filter paper. The whole root system was then covered with black plastic sheeting and grown under light for 2–4 h; after growth, the filter paper pieces attached to the surface of the root system were placed in a test tube and washed with sterile water, and the washing solution was collected to obtain the root exudates (Neumann et al., 1999; Zhongmin et al., 2000). By this method, the collected root exudates are closer to the natural state than in the hydroponic method. Still, the filter paper is easy to lose water and is of small adsorption capacity. The isotope labeling combined with the soil solution sampler is suitable for collecting exudates from large and small roots and reflects the *in situ* nature of root exudates. The principle is that the plants are fed with labeled isotopes, and then the rhizosphere solution is periodically extracted with the aid of a soil solution sampler; this solution is collected, filtered, and the resulting root exudates are extrapolated from the abundance of the labeled elements to the strength of the root exudates. This method of collecting root exudates is not very reproducible as the accurate distribution of the plant’s roots in the soil is unknown (Bienfait et al., 1989). In recent years, with the development of technology, greater breakthroughs have been made in collecting root exudates to a certain extent. In addition to the collection, real-time monitoring of root exudates can be achieved with minor disturbance. For example, micro-suction cups (MCSs) can monitor the average concentration of the inter-root soil solution, but it is still incompetent to quantify the composition (Puschenreiter et al., 2005).

## Conclusion

We synthesize current properties and regulators of root exudates and their role in the belowground ecosystem as substances cycle and signal to mediate. Plant species, growth stage, environmental factors, and microorganisms are central factors governing the composition and amount of root exudates and their production mechanism. Therefore, Root exudates play an important role in soil physicochemical properties, plant nutrient uptake, transformation and utilization, allelopathy, and environmental stress relief.

## Prospects

In this paper, we summarized the composition and amount of root exudates and their production mechanism are mainly driven by plant species, growth stage, environmental factors, and microorganisms. To further understand the mechanism of organic carbon and nitrogen cycling of root exudates in the underground ecosystem. Based on the results of this study, we believe that further research should be conducted in the future direction as follows.

(1) Plant species affect the amount of root exudates. The differences in physiological function, chemical composition, and morphological characteristics in different shrub root sequences can affect the root system. Therefore, future studies can refine the differences in strengthening shrubs at subtle scale differences of carbon and nitrogen inputs in root exudates and their relationship with the rhizosphere environment. For example, it can be collected through filter paper the root exudates of fine roots in different root orders of the alpine shrub were collected to identify the root exudates secretion of fine roots and different root segments in the same root order.
(2) At present, most of the studies on plant root C and N secretion mainly focus on the surface soil, but there are few studies on the deep soil. To further explore the mechanism of plant root carbon and nitrogen secretion, the study of deep root exudates should be strengthened in the future.
(3) The composition of root exudates is affected by multiple factors, so future studies can be conducted on the root exudates, aboveground vegetation types, and fine root functions in the process of shrub encroachment development were clarified the relationship among traits, environmental factors, and seasons, and the main factors regulating the components of root exudates were clarified to reveal the dynamic change characteristics and the response of root exudate components in the process of shrub encroachment and development mechanism.

## Author contributions

WM was responsible for writing guidance and manuscript preparation. ST was responsible for writing the first draft of the article. ZD was responsible for writing guidance. DZ, TZ, and XM were responsible for the revision of the article and the response of the review comments. All authors contributed to the article and approved the submitted version.
